# Supplementary material for: Distance to native climatic niche margins explains establishment success of alien mammals
Source: Nat Commun. 2021 Apr 21;12:2353. doi: 10.1038/s41467-021-22693-0 (PMC8060396; doi:10.1038/s41467-021-22693-0)
Supplement: Supplementary file 1 — Supplementary Information [file 41467_2021_22693_MOESM1_ESM.pdf]

Supplementary **Information**

Supplementary Notes 2

Note 1 – Influence of climatic suitability (CS) on establishment success 2

Note 2 – Influence of NMI on establishment success using phylogenetic regressions 3

Supplementary Figures 4

Figure 1– PCA results. 4

Figure 2 – Influence of NMI on establishment success for individual species. 5

Figure 3 – Phylogenetic logistic regressions. 6

Figure 4 – Schematic representation of NMI and CS as metrics of NCN-matching 7

Figure 5 – Detailed results of the Bayesian hierarchical mixed effect model for NMI. 8

Figure 6 – Posterior coefficients of the Bayesian models. 9

Figure 7 – Sensitivity analysis: climatic resolution and level of occurrence density 10

Figure 8 – Sensitivity analysis: Minimum volume ellipsoid (mve) 11

Figure 9 - Sensitivity analysis: posterior coefficients 12

Figure 10 - Sensitivity analysis: predictive accuracy 13

Figure 11 - NMI to realized niche vs. proxy of fundamental climatic niche 14

Supplementary References 15

Supplementary Notes

Supplementary Note 1 – Influence of climatic suitability (CS) on establishment success

To check the robustness of our results, we repeated our analyses by replacing the niche margin index (NMI) by a traditionally used climatic suitability measure (CS) obtained from species distribution models (SDMs[1]). SDMs were calibrated for all species using an ensemble modelling approach [2] implemented in the *biomod2* R package [3]. The ensemble included three different techniques: generalized linear models (GLM[4]), gradient boosting machines (GBM[5]) and maximum entropy (MAXENT[6]), which were implemented using the default settings of biomod2. The same set of climatic variables used for the PCA analyses were used to fit SDMs[7]: annual aridity (ai), precipitation of the driest quarter (pdryq), precipitation of the warmest quarter (pwarq), precipitation of the wettest quarter (pwetq), temperature of the coldest quarter (tcoldq), temperature of the warmest quarter (twarmq), daily range temperature (tdr), and temperature seasonality (ts). The extent of the geographic background was defined as the whole world to allow global extrapolation when projecting the models into new geographic areas. All pixels falling inside the native niche of a species were considered as presences (same dataset as for the native niche in the PCA). We randomly sampled 10,000 pseudo-absences within the geographic background, and weighted presences and absences in the models to ensure a prevalence of 0.5[8]. For each modeling algorithm, 10 iterations of the model were performed. The predictive performance was evaluated using a repeated split-sample approach, with 70% occurrence records used for training the model and 30% for evaluation. Models were evaluated using the true skill statistic (TSS[9]) across all possible thresholds between 0 and 1 (maxTSS[1]). The contribution of variables was evaluated through a permutation procedure: model predictions were recalculated with randomly reshuffled variables, and Pearson's correlation coefficient was calculated between the initial and reshuffled predictions; 1 minus the correlation provides the contribution of the variable, with 0 indicating that the variable has no influence on the model (function *variables_importance* in *biomod2*). The reshuffling procedure was performed 5 times and correlations were averaged. The overall accuracy of models was excellent, with a mean TSS among species of 0.986 ∓ 0.011 (SOM Table S1). The variable with the highest average contribution was precipitation in the warmest quarter (0.45 ∓ 0.163), and the one with the lowest average contribution was aridity (0.105 ∓ 0.087) (Supplementary Data 1).

We obtained similar results to NMI with CS (Wilcoxon test: W = 65253, $p<1x10^{-6}$, Bayesian model: Bayesian p-value=0.23; posterior P[effect of *CS*>0] = 100%).

Supplementary Note 2 – Influence of NMI on establishment success using phylogenetic regressions

Because it was not possible to implement the complete phylogenetic structure in the hierarchical Bayesian mixed effect model to account for the potential correlation between variables among and within species (because we used Bayesian imputation to deal with missing trait values), we also used phylogenetic logistic regression to test the association between establishment success and NMI.

We used the previously published phylogenetic tree of 5020 species of mammals[10] modified by [11,12]. All species in our dataset were present in the tree except for five species for which we chose the closest representative within the genus (*Equus grevyi* instead of *E. quagga*, *Callosciurus finlaysonii* instead of *C. erythraeus*, *Lama glama* instead of *L. guanicoe*, *Pseudocheirus peregrinus* instead of *P. occidentalis* and *Sciurus griseus* instead of *S. aberti*). We then grafted on this phylogenetic tree of 177 species, 114 individuals inside their respective species in order to provide a tip for each introduction (mean= 7.1 individuals per species, min=1, max= 35). Because the relationships between individuals inside species were not known *a priori*, we generated two distributions of 100 phylogenies. Each tip representing one individual was grafted on the terminal branch of the species given an age drawn from a uniform distribution (between 500 and 10000 years for the first distribution of 100 trees, and between 500 and 50000 years for the second distribution, consistently with ages found in the literature for mammals[13–16]). This grafting procedure was realized using the *multi2di* and the *bind.tip* functions in the R package *ape.*

We fitted a phylogenetic logistic regression described in[17] using the R package *phyloglm* between the introduction success and either NMI or CS. Introduction success was the response variable (binary), while niche innerness or climate suitability were the explanatory variables (continuous).The model was fitted for each tree of the two distributions of 100 phylogenies. Convergence was reached using maximized penalized likelihood of the logistic regression ("logistic_MPLE" using a Firth's correction) for both the association between success and NMI or CS. Wald type p-value for coefficients were obtained using parametric bootstrap based on 100 fitted replicates to test the null hypothesis that the regression coefficients differ from 0 (two-tailed test)

The detailed results of these analyses can be found in Supplementary Figure 3

Supplementary Figures

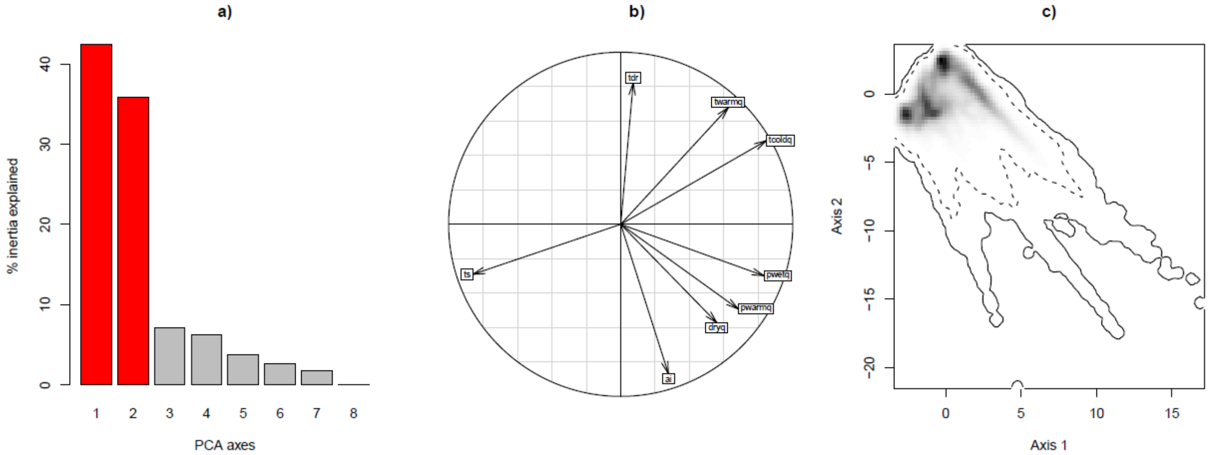

Supplementary Figure 1 – PCA results. a) Inertia explained by the axes of the Principal Component Analysis (PCA). The first and second axes explain 42.50 % and 35.83% of the inertia of the PCA, respectively. b) Correlation circle showing the contribution of initial variables to PCA axes (ai = aridity index, tdr = daily temperature range, ts = temperature seasonality, tcoldq = temperature of the coldest quarter, twarmq = temperature of the warmest quarter, pdryq = precipitation of the driest quarter, pcoldq = precipitation of the coldest quarter, pwarmq = precipitation of the warmest quarter) c) climatic space along the two first PCA axes with the density of climate worldwide in gray (black = most common climates).

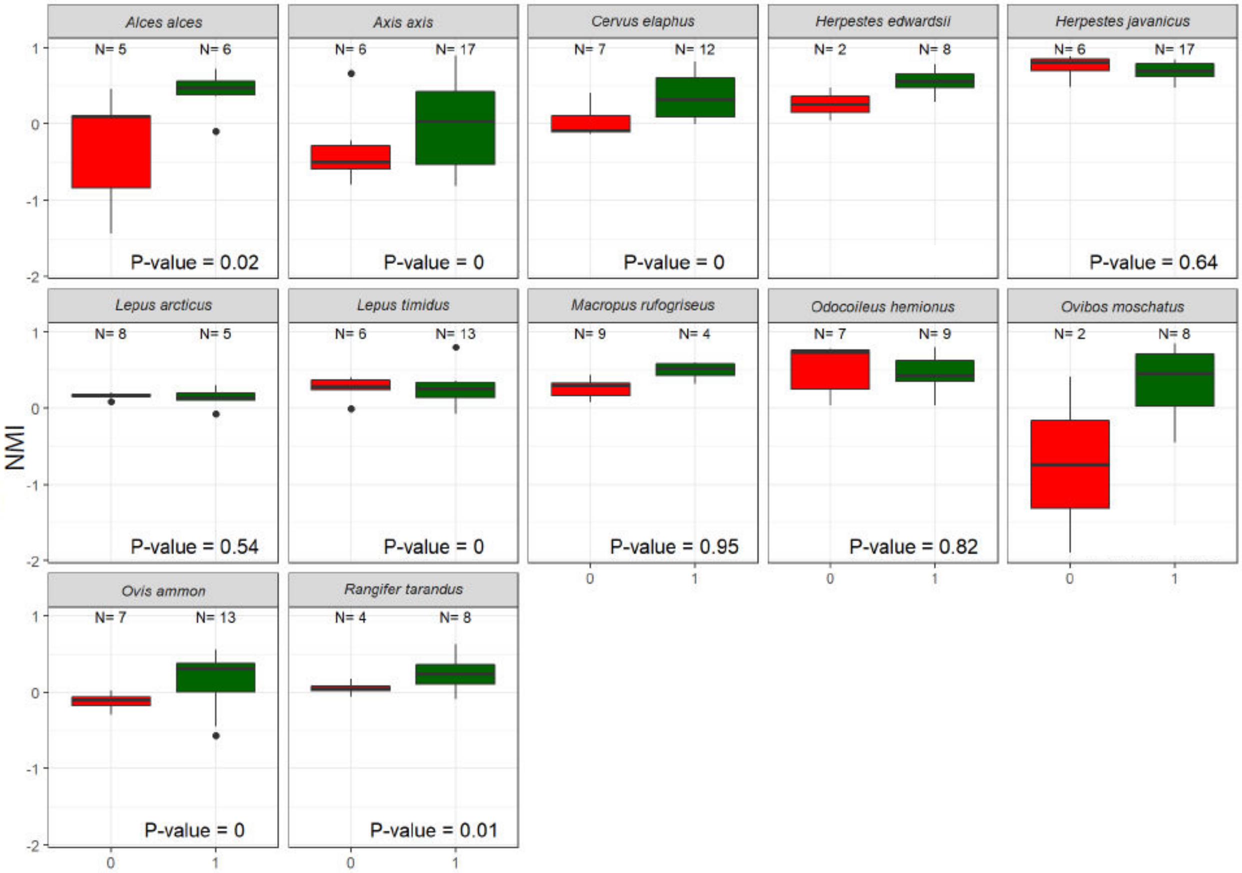

Supplementary Figure 2 – Influence of NMI on establishment success for individual species. Boxplots show the median (center line), the upper and lower quartiles (box limits), the 1.5x interquartile range (whiskers) of the niche margin index (NMI) for species with a number of introductions >9 and a ratio establishment success/ failure between 0.2 and 5. P-values are for Wilcoxon tests (two-tailed test). The sample size N is indicated for each species.

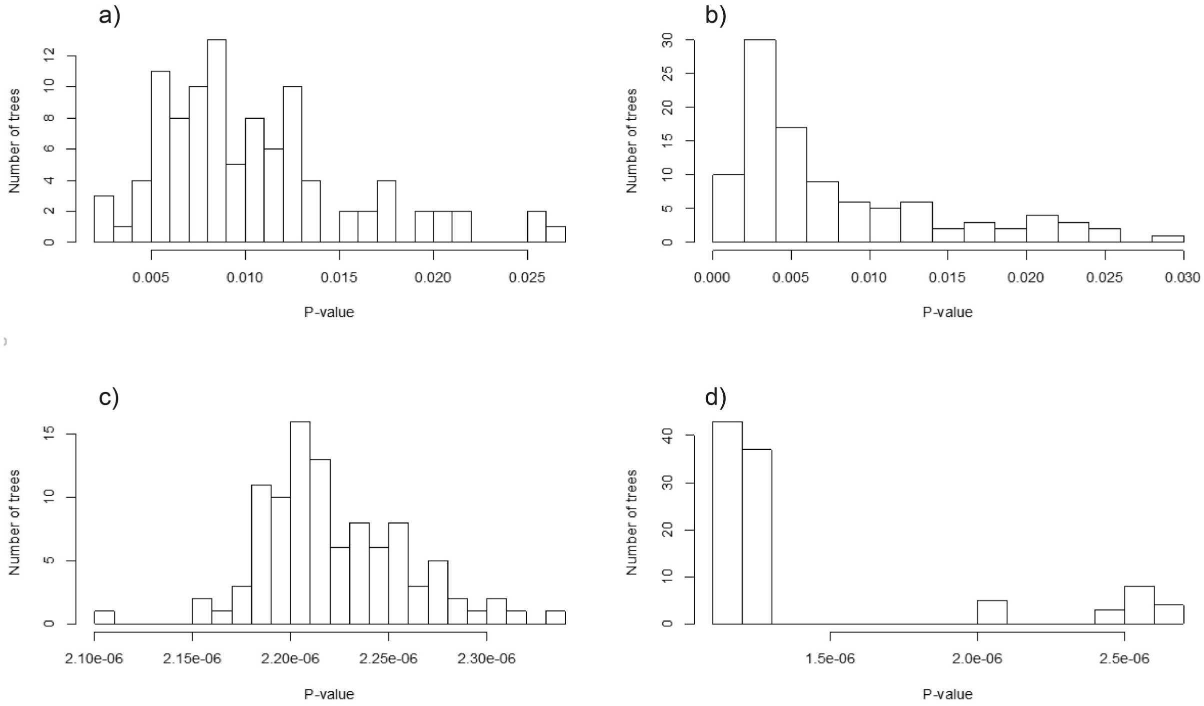

Supplementary Figure 3 – Phylogenetic logistic regressions. We tested the significance of the relationship between establishment success and the niche margin index (NMI; a,b), and climatic suitability (CS; c,d) using the function *phyloglm*[17] of the R package *phylolm*. Because the relationships between individuals inside species were not known, we generated two distributions of 100 phylogenies, with each tip representing one individual grafted on the branch of the species given an age drawn from a uniform distribution (between 500 and 10000 years for the first distribution of 100 trees (a, c), and between 500 and 50000 years for the second distribution (b, d), consistently with ages found in the literature for mammals[13-16] (See Supplementary Note 2). Phylogenetic logistic regressions were then fitted for each tree of the two distributions of 100 phylogenies. Wald type p-value for coefficients were obtained using parametric bootstrap based on 100 fitted replicates to test the null hypothesis that the regression coefficients differ from 0 (two-tailed test). We plot here the distributions of the 100 p-value obtained for the two distributions of 100 trees for the two explanatory variables. No correction was applied for multiple testing. In all simulations, the slope of the regression between the establishment success and each of its predictor variables is significantly different from 0 (p-value < 0.05).

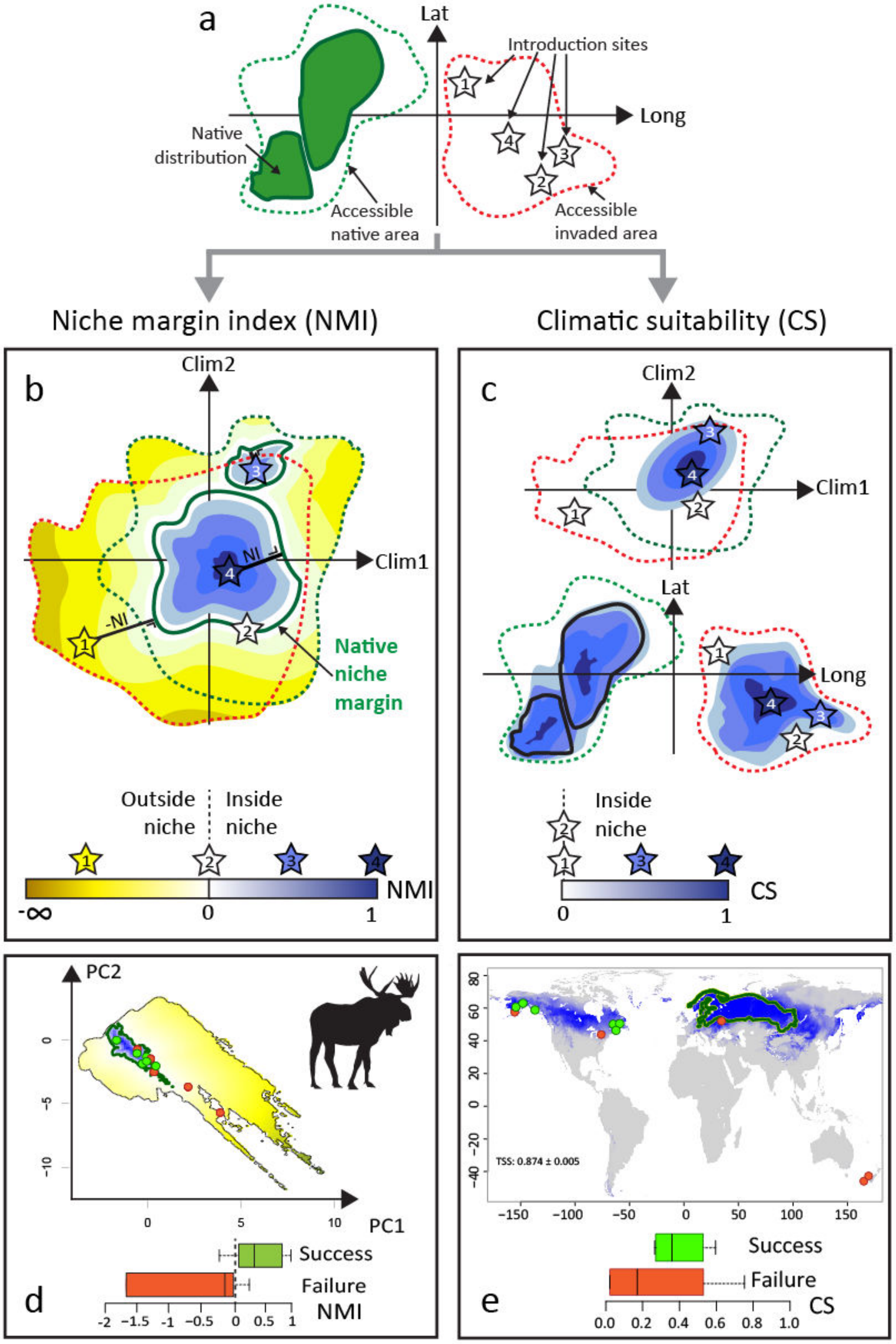

Supplementary Figure 4 – Schematic representation of NMI and CS as metrics of NCN-matching. (a) native distribution, accessible areas (sensu Barve et al. 2011) and exotic introduction in geographical space. (b) Schematic representation of the niche margin index (NMI) with distances of introduction sites to native niche margins in climatic space. (c) Climatic suitability (CS) of introduction sites in climatic and geographic space. Introductions outside the native niche are floored to 0 for CS. (d) and (e) Illustration of NMI and CS for the Elk (*Alces alces*). Boxplots include the median (center line), the upper and lower quartiles (box limits), the 1.5x interquartile range (whiskers). N = 11 independent introductions. Drawings and schematic representations are made by the authors.

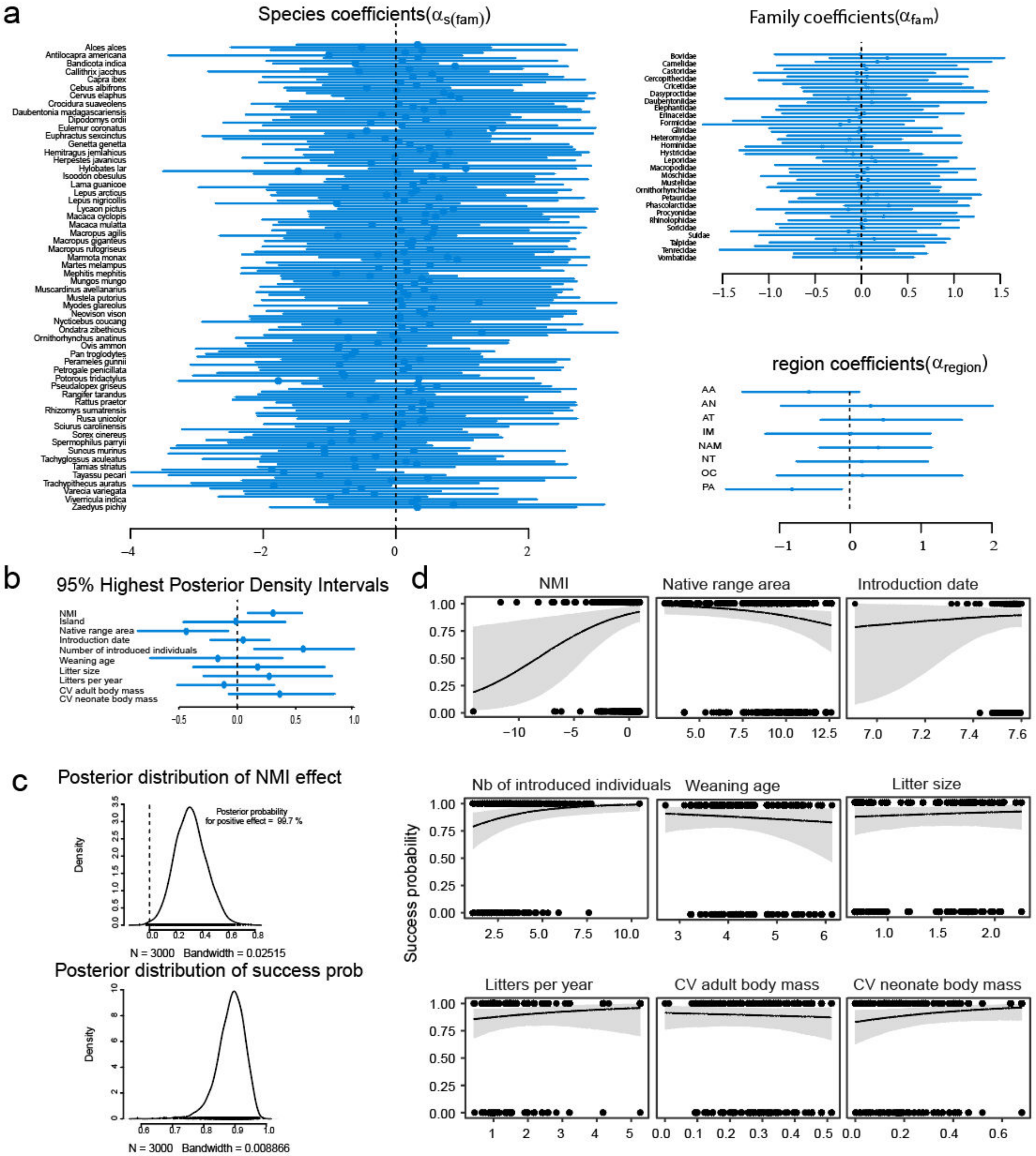

Supplementary Figure 5 – Detailed results of the Bayesian hierarchical mixed effect model with NMI as the response variable. In all panels, inferences were derived using n=3000 independent estimates sampled from the joint posterior distribution. (a) Posterior estimates (logit scale) of random factors (i.e. species-wise, family-wise and region-wise intercepts) with blue points representing the medians and blue lines representing the corresponding 95% highest posterior density (HPD) intervals. The vertical dashed line points to the zero value. (b) Posterior estimates (logit scale) of fixed factors with blue points representing the median and blue lines representing the corresponding. The vertical dashed line points to the zero value. (c) Posterior distribution of the effect of NMI on establishment success (top panel) and of the estimated probability of successful establishment (bottom panel). (d) Response curves showing the effect of fixed factors on the probability of establishment success. Solid lines and grey areas show the median of the posterior distribution of effects and the associated 95% highest posterior density intervals, respectively. Black points show the observed data, n = 979 independent introductions.

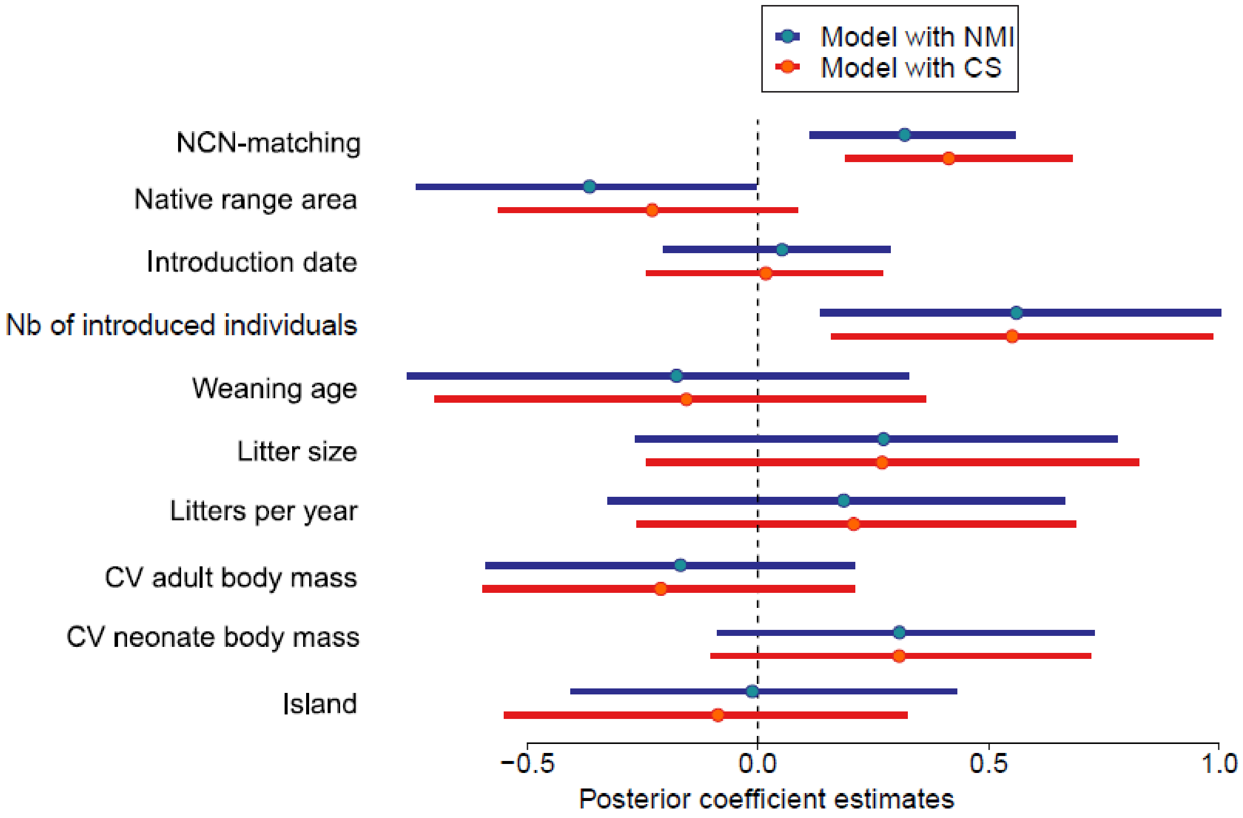

Supplementary Figure 6 – Posterior coefficients of the Bayesian models. Horizontal bars represent the 95% Highest Posterior Density (HPD) intervals for fixed effects in the model calibrated with NMI (in blue) and the model calibrated with CS (in red). Dots represent the median of the posterior distribution of effects. The vertical dotted line indicates no effect. N = 3000 independent samples from the posterior distribution of model estimates.

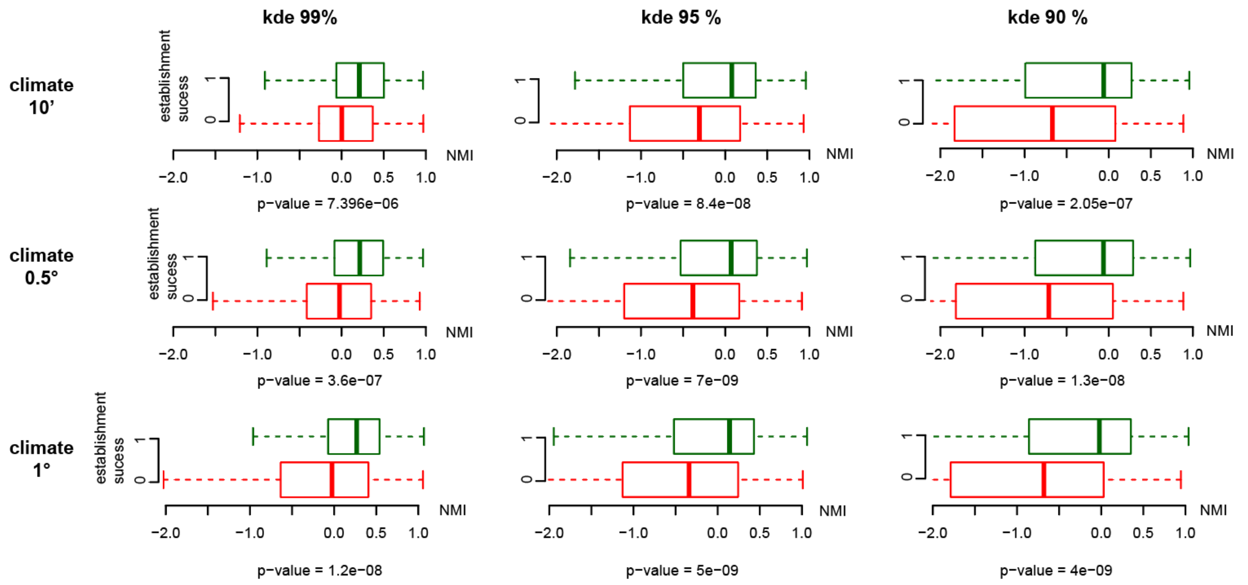

Supplementary Figure 7 – Sensitivity analysis on the climatic resolution and level of occurrence density. The boxplots represent the NMI values obtained for successful and failed establishment for each combination of resolution of the climatic dataset (10min, 0.5°, 1°) and level of estimated density of occurrence in the kernel density estimation (kde; 99%, 95%, 90%). P-values below the plots indicate the results of a Wilcoxon rank sum test (two-tailed test). N = 979 independent introductions. Overall the results show that successful introductions have significantly higher NMI values than failed ones under all settings, but that NMI values increase with the level of density of the kde. The resolution of the climatic dataset does not have a strong influence on the results.

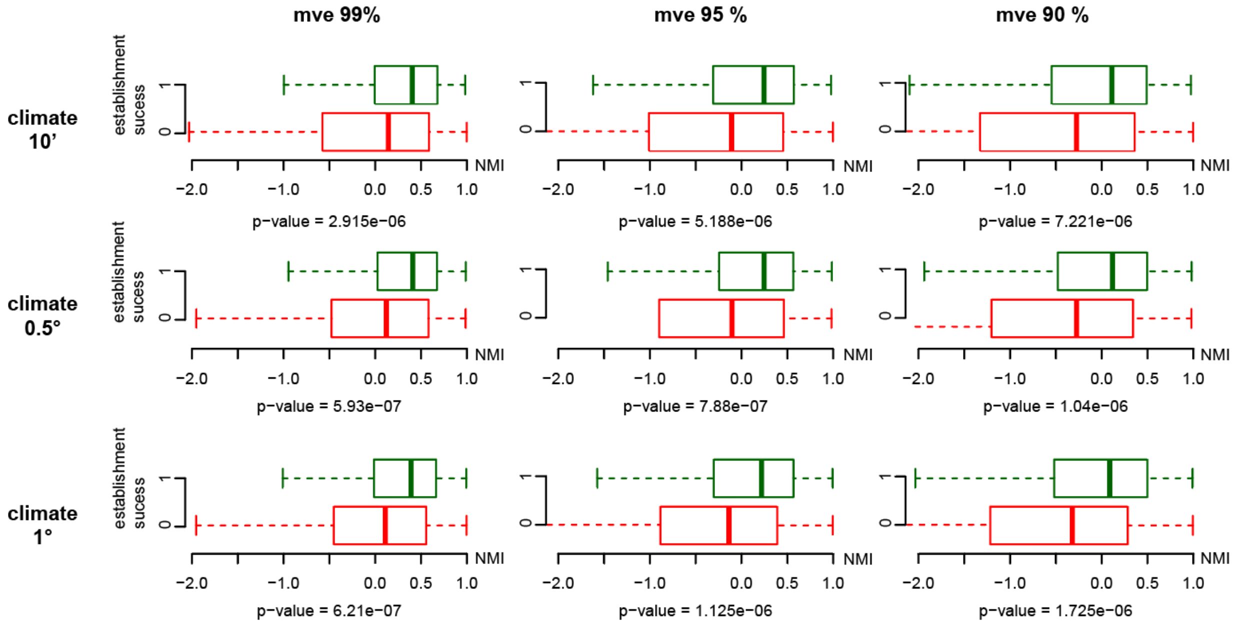

Supplementary Figure 8 – Sensitivity analysis on the minimum volume ellipsoid (mve). Same boxplots as Figure S7 but based on mve instead of kernel density estimation (kde) envelopes. Sensitivity to the resolution of the climatic dataset (10min, 0.5°, 1°) and level of inclusion of occurrence in the mve (99%, 95%, 90%) are shown. P-values below the plots indicate the results of a Wilcoxon rank sum test (two-tailed test). N = 979 independent introductions. Similarly to kde results, we show that successful introductions have significantly higher NMI values than failed ones under all settings, but that NMI values increase with the level of density of the mve. The resolution of the climatic dataset does not have a strong influence on the results

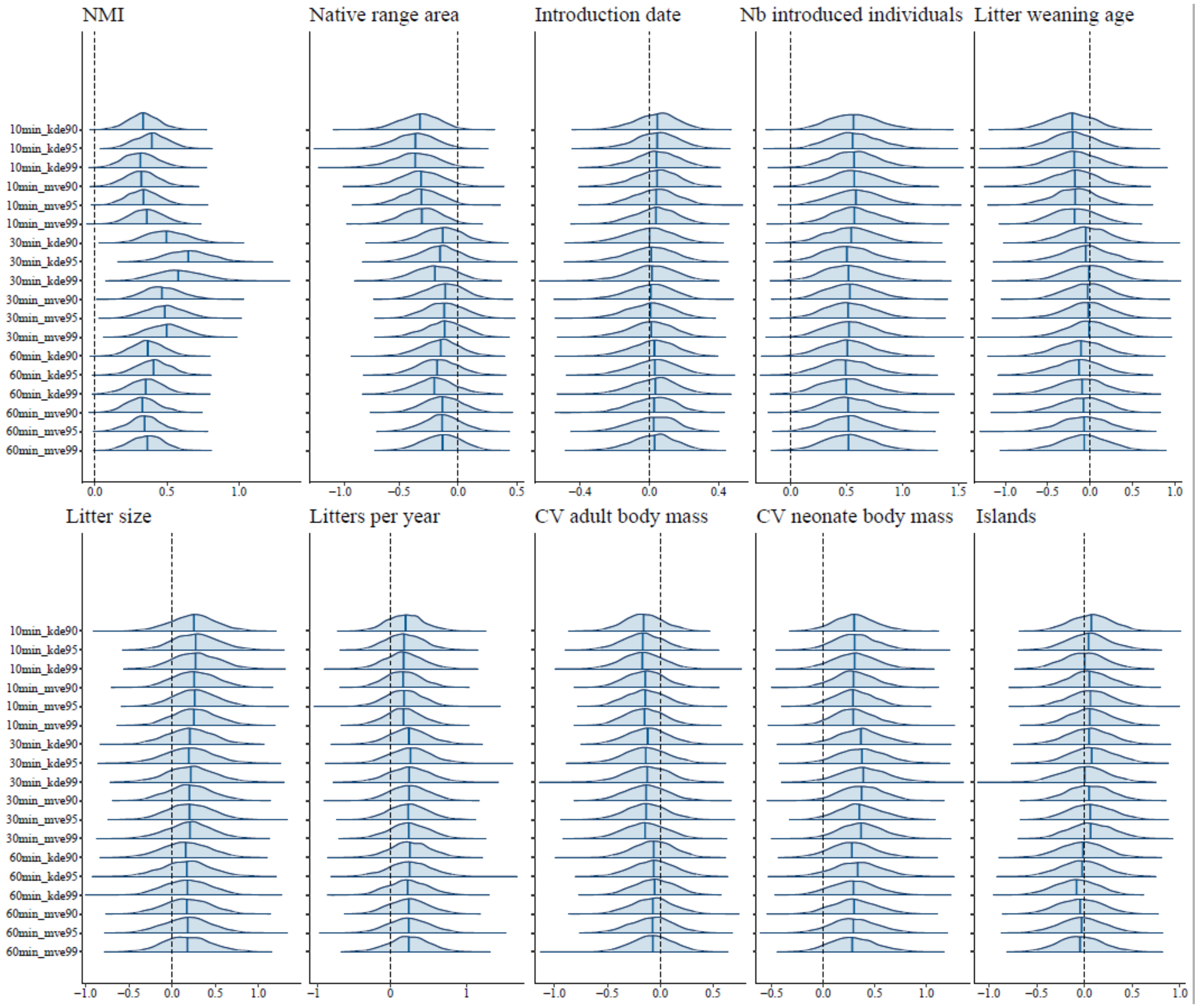

Supplementary Figure 9 – Sensitivity analysis: posterior coefficients. Posterior distribution of coefficients of fixed effects across all climatic resolutions (10',0.5°,1°), levels of density (99%,95%,90%) and type of niche margin envelopes (kernel density estimation, kde; minimum volume ellipsoid, mve). Vertical blue lines represent the median of the posterior distribution of effects (i.e. the strength of effect), while the blue shaded areas under the curves represent the 95% Highest Posterior Density (HPD) intervals. The vertical dotted line indicates no effect. N = 3000 independent samples from the posterior distribution of model estimates.

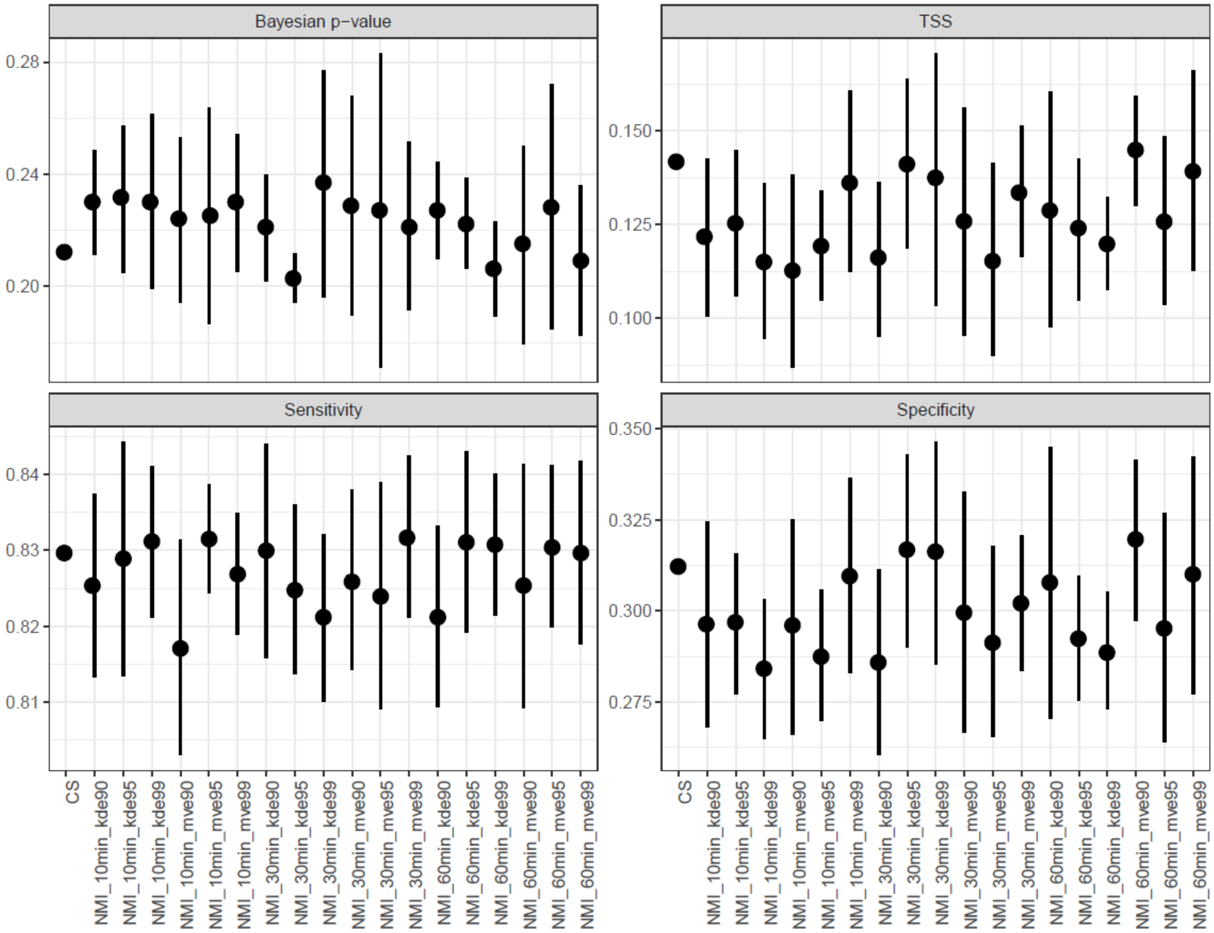

Supplementary Figure 10 – Sensitivity analysis: predictive accuracy. Median (point) and associated 95% Highest Posterior Density (HPD) intervals (vertical bars) of Bayesian p-values, True Skill Statistic (TSS), sensitivity and specificity of Bayesian models obtained from the repeated split-sample procedure performed (see methods) across all climatic resolutions (10',0.5°,1°), levels of density (99%,95%,90%) and type of niche margin envelopes (kernel density estimation, kde; minimum volume ellipsoid, mve). The Bayesian p-values are posterior predictive checks calculated from the sum of squared standardized Pearson residuals for both the observed data and a replicated dataset derived from model estimates (see methods). Bayesian p-values quantify the proportion of samples in which the distance of observed data to the model is greater than the distance of replicated data to the model. Values close to 0.5 suggest a good model fit, whereas values close to 0 or 1 indicate a lack of fit. N = 3000 independent samples from the posterior distribution of model estimates. For comparison, the values obtained on the full dataset for the models based on NMI and CS are shown on the left of each panel.

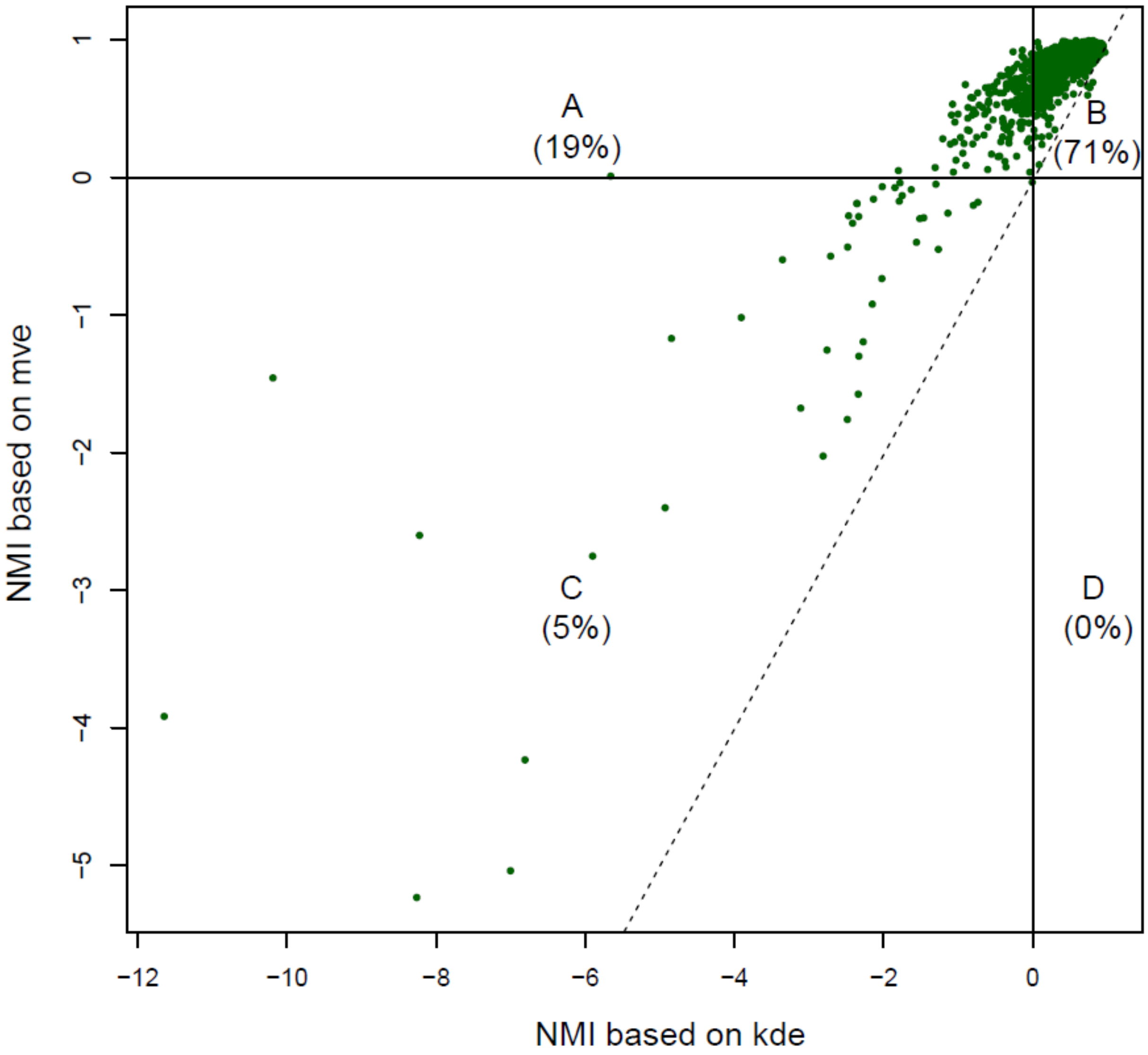

Supplementary Figure 11 – NMI to realized niche vs. proxy of fundamental climatic niche. NMI for all successful establishments are shown in green in the plot, with the NMI calculated with the kernel density estimation (kde) approach on the x-axis and the NMI calculated with the minimum volume ellipsoid (mve) approach on the y-axis. N = 787 independent successful establishments. According to classical niche theory, successful establishments should only be located in the regions B (inside the NCN) or A (outside the NCN but inside the fundamental climatic niche, owing to competitive release in the exotic range). Region C corresponds to successful establishments outside the fundamental niche (i.e. the tolerance niche[18]).

Supplementary References

1. Guisan, A., Thuiller, W. & Zimmermann, N. E. *Habitat Suitability and Distribution Models: with Applications in R*. (Cambridge University Press, 2017).
2. Araujo, M. & New, M. Ensemble forecasting of species distributions. *Trends Ecol. Evol.* **22**, 42–47 (2007).
3. Thuiller, W., Lafourcade, B., Engler, R. & Araújo, M. B. BIOMOD - a platform for ensemble forecasting of species distributions. *Ecography* **32**, 369–373 (2009).
4. McCullagh, P. & Nelder, J. A. *Generalized Linear Models*. (1989).
5. Friedman, J. H. machine. *Ann. Stat.* **29**, 1189–1232 (2001).
6. Phillips, S. J., Anderson, R. P. & Schapire, R. E. Maximum entropy modeling of species geographic distributions. *Ecol. Modell.* **190**, 231–259 (2006).
7. Petitpierre, B., Broennimann, O., Kueffer, C., Daehler, C. & Guisan, A. Selecting predictors to maximize the transferability of species distribution models: lessons from cross-continental plant invasions: Which predictors increase the transferability of SDMs? *Glob. Ecol. Biogeogr.* **26**, 275–287 (2017).
8. Barbet-Massin, M., Jiguet, F., Albert, C. H. & Thuiller, W. Selecting pseudo-absences for species distribution models: how, where and how many? *Methods Ecol. Evol.* **3**, 327–338 (2012).
9. Allouche, O., Tsoar, A. & Kadmon, R. Assessing the accuracy of species distribution models: prevalence, kappa and the true skill statistic (TSS). *J. Appl. Ecol.* **43**, 1223–1232 (2006).
10. Bininda-Emonds, O. R. P. *et al.* The delayed rise of present-day mammals. *Nature* **446**, 507–512 (2007).
11. Fritz, S. A., Bininda-Emonds, O. R. P. & Purvis, A. Geographical variation in predictors of mammalian extinction risk: big is bad, but only in the tropics. *Ecol. Lett.* **12**, 538–549 (2009).
12. Kuhn, T. S., Mooers, A. Ø. & Thomas, G. H. A simple polytomy resolver for dated phylogenies. *Methods Ecol. Evol.* **2**, 427–436 (2011).
13. Rohde, D. L. T., Olson, S. & Chang, J. T. Modelling the recent common ancestry of all living humans. *Nature* **431**, 562–566 (2004).
14. Freedman, A. H. *et al.* Genome sequencing highlights the dynamic early history of dogs. *PLoS Genet.* **10**, e1004016 (2014).
15. Rajabi-Maham, H., Orth, A. & Bonhomme, F. Phylogeography and postglacial expansion of Mus musculus

domesticus inferred from mitochondrial DNA coalescent, from Iran to Europe. *Mol. Ecol.* **17**, 627–641 (2008).

16. Fernandez, H. *et al.* Divergent mtDNA lineages of goats in an Early Neolithic site, far from the initial domestication areas. *Proceedings of the National Academy of Sciences* **103**, 15375–15379 (2006).

17. Ives, A. R. & Garland, T., Jr. Phylogenetic logistic regression for binary dependent variables. *Syst. Biol.* **59**, 9–26 (2010).

18. Sax, D. F., Early, R. & Bellemare, J. Niche syndromes, species extinction risks, and management under climate change. *Trends Ecol. Evol.* **28**, 517–523 (2013).
