# Supplementary material for: Distance to native climatic niche margins explains establishment success of alien mammals
Source: Nat Commun. 2021 Apr 21;12:2353. doi: 10.1038/s41467-021-22693-0 (PMC8060396; doi:10.1038/s41467-021-22693-0)
Supplement: Supplementary file 3 — Description of Additional Supplementary Files [file 41467_2021_22693_MOESM3_ESM.pdf]

Description of Additional Supplementary Files

Title: Supplementary Data 1

Description: Species-wise SDM results. The first two columns show the evaluation metrics by TSS. The following columns provide the contribution to the models (see methods) of the following variables: annual aridity (ai), precipitation of the driest quarter (pdryq), precipitation of the warmest quarter (pwarq), precipitation of the wettest quarter (pwetq), temperature of the coldest quarter (tcoldq), temperature of the warmest quarter (twarmq), daily range temperature (tdr), and temperature seasonality (ts).
