# Supplementary material for: Distance to native climatic niche margins explains establishment success of alien mammals
Source: Nat Commun. 2021 Apr 21;12:2353. doi: 10.1038/s41467-021-22693-0 (PMC8060396; doi:10.1038/s41467-021-22693-0)
Supplement: Supplementary file 5 — Reporting Summary [file 41467_2021_22693_MOESM5_ESM.pdf]

nature research

Corresponding author(s): Olivier Broennimann

Last updated by author(s): Mar 9, 2021

Reporting Summary

Nature Research wishes to improve the reproducibility of the work that we publish. This form provides structure for consistency and transparency in reporting. For further information on Nature Research policies, see our Editorial Policies and the Editorial Policy Checklist.

Statistics

For all statistical analyses, confirm that the following items are present in the figure legend, table legend, main text, or Methods section.

n/a	Confirmed	
☐	☒	The exact sample size (n) for each experimental group/condition, given as a discrete number and unit of measurement
☐	☒	A statement on whether measurements were taken from distinct samples or whether the same sample was measured repeatedly
☐	☒	The statistical test(s) used AND whether they are one- or two-sided<br>*Only common tests should be described solely by name; describe more complex techniques in the Methods section.*
☐	☒	A description of all covariates tested
☐	☒	A description of any assumptions or corrections, such as tests of normality and adjustment for multiple comparisons
☐	☒	A full description of the statistical parameters including central tendency (e.g. means) or other basic estimates (e.g. regression coefficient) AND variation (e.g. standard deviation) or associated estimates of uncertainty (e.g. confidence intervals)
☐	☒	For null hypothesis testing, the test statistic (e.g. F, t, r) with confidence intervals, effect sizes, degrees of freedom and P value noted<br>*Give P values as exact values whenever suitable.*
☐	☒	For Bayesian analysis, information on the choice of priors and Markov chain Monte Carlo settings
☒	☐	For hierarchical and complex designs, identification of the appropriate level for tests and full reporting of outcomes
☐	☒	Estimates of effect sizes (e.g. Cohen's d, Pearson's r), indicating how they were calculated

Our web collection on statistics for biologists contains articles on many of the points above.

Software and code

Policy information about availability of computer code

Data collection	no software was used
Data analysis	R version 4.0.3, JAGS 4.3.0, code available on github.com/ecospat/NMI

For manuscripts utilizing custom algorithms or software that are central to the research but not yet described in published literature, software must be made available to editors and reviewers. We strongly encourage code deposition in a community repository (e.g. GitHub). See the Nature Research guidelines for submitting code & software for further information.

Data

Policy information about availability of data

All manuscripts must include a data availability statement. This statement should provide the following information, where applicable:

- Accession codes, unique identifiers, or web links for publicly available datasets
- A list of figures that have associated raw data
- A description of any restrictions on data availability

The introduction dataset, including information about establishment success, life-history traits, historical factors and release characteristics, is available on github.com/ecospat/NMI. Raw data on establishment success is shown in Fig. 2a. We now provide a supplementary Source Data to replicate Fig. 1 and 2.

Field-specific reporting

Ecological, evolutionary & environmental sciences study design

All studies must disclose on these points even when the disclosure is negative.

Study description	We modeled the establishment success of invasive mammals as a function of the matching to the native climatic niche (NCN), life-history traits and historical factors at the release-event level.
Research sample	Introduction events of invasive mammals worldwide digitalized from Long et al. 2003
Sampling strategy	We recorded the geographic coordinates, date and outcome of all introduction events from Long et al. 2003 for species with fewer than 20 known introductions and the 20 first for species with more than 20 introductions. This assessment was based on the description of the species in the IUCN Red List database (iucnredlist.org; accessed in November 2013), in particular on information from the “range description” section. Reintroductions in historical parts of the native range of species were not considered. Only species that had no major documented contractions of their range extent during historical times were considered for analysis (but range fragmentations were allowed). Only populations that were explicitly described as established or expanding were considered as successfully established. It resulted in a database of 989 introduction events for 177 mammal species, with geographical coordinates and indicating whether the establishment was successful or failed.
Data collection	For every introduction we calculated or gathered information about 1) matching to the native climatic niche (NCN), 2) historical factors at the release-event level, and 3) life-history attributes at the species level commonly considered in this context.<br>- matching to NCN was measured as the minimum distance to the margin of the NCN along the first two axes of a principal component analysis including eight bioclimatic variables at a resolution of 30’’. The native ranges of mammal species were extracted from the IUCN Red List database (iucnredlist.org) and we considered areas labeled as “extant”, “probably extant”, “reintroduced”, “probably extinct” and “extinct” as part of the native range. For each species, we delimited the NCN by using the scores of the pixels in the PCA space belonging to the native niche according to the IUCN.<br>- Historical factors included introduction date (digitized from Long et al. 2003), propagule pressure (digitized from Long et al. 2003), introduction on mainland vs. island (extracted from GADM data shapefile; gadm.org), native range area (extracted from IUCN Red List database shapefile; iucnredlist.org) and the biogeographic region of introduction (extracted from the shapefile of terrestrial ecoregions; maps.tnc.org/gis_data.html).<br>- Life-history traits included litter weaning age, litter size, number of litters per year, coefficient of variation of adult body mass and coefficient of variation of neonate body mass (Gonzalez-Suarez 2015).
Timing and spatial scale	The status of failure or success of introduction points we recorded between 1969 and 2000 in Long et al. 2003. The digitalization of the geographic coordinates was done in ArcGIS by matching the descriptions in Long et al. 2003 with GADM data, google map, and other available administrative boundary data. The coordinates were recorded with a precision below the resolution of the climatic data (10' =~16km).
Data exclusions	No data were excluded from the analyses
Reproducibility	All the data used were either digitalized from the book written by Long et al. or extracted from published databases using automatized procedures. Our work is thus fully reproducible.
Randomization	Because we digitalized all known introduction events from a book, they were not randomly allocated in groups. We did control for covariates by using species and biogeographic regions as random factors.
Blinding	Our study involves only modeling approaches. No experimental data were collected, so blinding is not relevant.

Did the study involve field work? ☐ Yes ☒ No

Reporting for specific materials, systems and methods

We require information from authors about some types of materials, experimental systems and methods used in many studies. Here, indicate whether each material, system or method listed is relevant to your study. If you are not sure if a list item applies to your research, read the appropriate section before selecting a response.

Materials & experimental systems

n/a	Involved in the study	
☒	☐	Antibodies
☒	☐	Eukaryotic cell lines
☒	☐	Palaeontology and archaeology
☒	☐	Animals and other organisms
☒	☐	Human research participants
☒	☐	Clinical data
☒	☐	Dual use research of concern

Methods

n/a	Involved in the study	
☒	☐	ChIP-seq
☒	☐	Flow cytometry
☒	☐	MRI-based neuroimaging
